# Effect of Gig Workers’ Psychological Contract Fulfillment on Their Task Performance in a Sharing Economy—A Perspective from the Mediation of Organizational Identification and the Moderation of Length of Service

**DOI:** 10.3390/ijerph17072208

**Published:** 2020-03-25

**Authors:** Wenlong Liu, Changqing He, Yi Jiang, Rongrong Ji, Xuesong Zhai

**Affiliations:** 1 College of Economics and Management, Nanjing University of Aeronautics and Astronautics, Nanjing 211106, China; willenliu@nuaa.edu.cn (W.L.); changqinghe@nuaa.edu.cn (C.H.); m18015957645@163.com (Y.J.); jirongrong94@163.com (R.J.); 2 School of Management, Fudan University, Shanghai 200433, China; 3 College of Education, Zhejiang University, Hangzhou 310058, China

**Keywords:** sharing economy, gig employment, psychological contract fulfillment, organizational identification, task performance, length of service

## Abstract

Workers’ isolation may occur in gig employment in the sharing economy, which generates a weak perception of the organization and unpredictable work performance. Drawing on social exchange theory, this paper proposes a framework to explore the effect of psychological contract fulfillment on gig workers’ task performance from the perspective of the mediation of organizational identification and the moderation of the length of service. A total of 223 samples were recruited from Didi (a ride-hailing company in China) drivers. The results show that both transactional and relational psychological contract fulfillment can directly affect gig workers’ task performance and also indirectly affect it via organizational identification. When the length of service for the current company is taken into consideration, transactional contract fulfillment, as the representation of a company’s recognition of gig workers’ effort, has a stronger effect on the organizational identification of gig workers who have been working for the company for less than a year compared with those who have been working for a longer period. The results show no difference in the relationship between relational psychological contract and organizational identification between the two groups. Transactional psychological contract fulfillment exhibits the same significant effect on gig workers’ task performance in both groups. By contrast, relational psychological contract fulfillment has a stronger effect on long-serving Didi drivers than on those who joined the company within the year. These findings generate certain theoretical and practical implications for gig employment management in the sharing economy.

## 1. Introduction

The growth of information technology has generated various new business patterns and employment options. The emergence of “gig” and “sharing” economies in this trend enables companies to access the workforce in the cloud [1]. Organizations in various forms have benefited significantly from gig employment [2,3]. As one category of the gig economy, the sharing economy refers to companies that use online platforms to connect the supply and demand for services. Uber, Lyft, Didi, and Airbnb are typical sharing economy companies in accommodation or ride sharing. Compared with traditional full-time or permanent employment, gig jobs are generally conducted in online settings, which may result in workers’ isolation from each other and a sense of insecurity on the job, thereby threatening job outcomes [3,4].

A few studies have investigated gig workers’ perceived work-based social support, engagement, and job satisfaction [3,4,5], whereas workers’ performance under gig employment has rarely been discussed. Nevertheless, it has been attracting researchers’ attention to explore the driving mechanism of workers’ performance from varied perspectives under traditional employment, from which psychological contract fulfillment (PCF) was regarded as a vital predictor of task performance [6,7]. Psychological contracts reflect individuals’ beliefs about the terms and conditions of a reciprocal exchange agreement between individuals and the organization [8]. Transactional and relational contracts have been regarded as two main dimensions of psychological contracts [9,10]. The transactional psychological contract captures the organization’s provision of adequate monetary or economic compensation, working conditions, and reasonable guarantees of employment in exchange for employees’ fulfillment of their work obligations [9,11]. The relational psychological contract captures the organization’s provision of work-related training, professional development, fair treatment, and job security [10]. The fulfillment of psychological contracts will increase employees’ trust in the organization, which in turn contributes to employees’ attitudes and behaviors, including their commitment, satisfaction, and task performance [6,10,12]. In the sharing economy, gig workers face both economic and career instability. Arousing workers’ affective commitment and work engagement in the context of gig employment is one of the most pressing challenges for sharing economy companies. To address this problem, this paper proposes that PCF, including transactional and relational contract fulfillment, is positively linked to gig workers’ performance.

Meanwhile, organizational identification is an important factor in the web-based sharing economy because it facilitates gig workers’ cooperation and coordination with the organization [4]. Organizational identification is caused by individuals perceiving themselves as parts of an organization [13]. Thus, an important aspect of organizational identification is cognitive and depends on the salience of an individual’s perceived membership or affiliation with the organization [4]. The need for affiliation is a personality attribute corresponding to individuals’ desire for social contact or belongingness [14]. Belongingness may strengthen willingness and motivation to increase effort and consequently influence a worker’s level of engagement [15]. Gig workers’ need for affiliation and connection is demonstrated to positively influence organizational identification and commitment [4], thereby influencing their task performance [5,16]. Prior studies have determined that organizational identification serves as an important mediator between psychological contracts and employees’ in-role performance and service-oriented organizational citizenship behavior [11,17]. Drawing on these studies, this paper suggests that organizational identification will, to some extent, mediate the effect of PCF on gig workers’ task performance.

In addition, employees exhibit different expectations of their organization as their tenure increases [18]. Prior studies have found that the relationships between PCF and employee commitment, work engagement, and performance dynamically evolve with increasing service time [19,20,21]. In other words, organizational tenure moderates the relationships between PCF and employees’ job outcomes. Specifically, it is found that PCF is longitudinally related to higher work engagement and lower turnover intentions, but only for employees with low tenure [19]. Organizational tenure is also found to moderate the relationship between perceived PCF and employees’ performance such that the relationship is weaker for individuals with longer organizational tenure. Based on these studies, a gig worker’s tenure in the current company is proposed to moderate the relationship between PCF and organizational identification, as well as task performance.

Despite the growing scholarly interest over recent years, the discussion on gig employment in a sharing economy remains in its infancy. Gig workers who work independently outside organizations engage in a style of work that differs from that assumed by organizational behavior theories. Accordingly, Ashford et al. (2018) proposed an agenda for studying individuals in a gig economy to address those challenges that gig workers and organizations may confront, such as remaining viable, staying organized, maintaining identity, and sustaining relationships [22]. A recent review article by Jabagi et al. (2019) proposed that the basic needs satisfaction of a platform-based worker is positively related to his/her intrinsic work motivation [16]. For example, a platform with social features may enhance the perceived relatedness of a gig worker. While studies on gig employment in a sharing economy have mostly addressed the foreseeable problems that gig organizations may confront and inferred possible solutions, a deeper exploration into these propositions is warranted. When independent workers perform their jobs outside traditional centralized offices and conventional work hours, organizations cannot rely on managerial supervision as a means of controlling and motivating these workers [4,16]. Therefore, how to motivate gig workers to advance in fulfilling their tasks and achieving their organizational goals becomes an important issue. To address this problem, this paper has put forward several propositions as specified earlier in this section from the perspective of social exchange theory (SET) to understand the mechanism behind the influence of PCF on the attitudinal and behavioral outcomes of gig workers. The following questions were formulated for this purpose:

Question 1. What are the relationships between PCFs and gig workers’ task performance?

Question 2. Can organizational identification play a mediating role in these relationships?

Question 3. Do these relationships vary along with the gig workers’ service length for their current company?

To empirically examine these relationships, an interview-based survey was conducted among Didi (a ride-hailing company in China) drivers, and a structural equation modeling approach was used to test the hypotheses. The findings provide several theoretical and practical implications for research on gig employment and contribute to the effective management and sustainable development of sharing economy companies.

## 2. Theoretical Background and Hypotheses Development

### 2.1. Psychological Contract Theory

Psychological contracts have been discussed for decades in studies regarding employment relationships and employees’ attitudes and behaviors [23,24,25]. This concept was introduced by using the term “psychological work contract” to describe an implicit understanding between a group of employees and their foreman due to a particular leadership style [26]. On this basis, Levinson et al. (1962) defined psychological contracts as “a series of mutual expectations of which the parties to the relationship may not themselves be dimly aware but which nonetheless govern their relationship to each other” [27]. The definitions showed only slight differences among psychological contract studies in the early years [28]. However, Rousseau (1989, 1995) and Rousseau and Tuoriwala (1998) argued that the expectations in previous definitions could only be comprehended with difficulty. Thus, these authors proposed a relatively narrow definition from the perspective of the individual as the central element and defined the psychological contract as “individual’s beliefs, shaped by the organization, regarding terms of an exchange agreement between individuals and their organization (or another person)” [9,29,30]. Unlike the legal employment contract, the psychological contract exhibits a subjective character because it is implicit but relies on individuals’ perception regarding reciprocal obligations between themselves and their employing organization [6,31]. Researchers typically account for the mechanisms underlying psychological contracts using SET [32] because its central concept is the norm of reciprocity [33].

Psychological contract theory introduces two distinctive forms of psychological contracts in employment relations, namely, the transactional psychological contract and the relational psychological contract [9]. The key differences between the two forms of psychological contracts include the duration of the employment arrangement (short-term vs. open-ended), the degree of specificity (highly vs. loosely specified), the exchange of resources (tangible vs. intangible), and performance-reward contingencies (highly contingent vs. low or noncontingent) [17,34]. The transactional psychological contract refers to a short duration or economic, monetary, or materialistic emphasis on individuals’ reciprocal exchange agreement with their employers [34]. Employees with a strong transactional psychological contract primarily perceive their organization as a source of income [35]. In contrast, the relational psychological contract is long-term and broad and includes socio-emotional exchanges, mutual trust, and loyalty [36,37], such as long-term job security and career development [38]. Although later researchers distinguished psychological contracts into multiple dimensions, the most accepted approach to understanding the distinctive forms of psychological contracts is the two-dimensional approach, that is, transactional and relational psychological contracts [24,39,40].

Psychological contract-related studies focus mainly on three aspects, namely, obligations, fulfillment, and breaches. Obligations refer to employees’ perception of their employers’ specific promises, and PCF is the extent of employers’ fulfillment of their obligations based on employees’ perception [8]. In contrast, when employees perceive that their employer fails to keep its promises in the psychological contract, it is understood as psychological contract breach [36,41]. Obligations and fulfillment have likewise been proven to induce job satisfaction, organizational commitment, and low intention to quit [24,42]. In particular, PCF has been linked to employees’ performance more strongly than obligations [43], given that repeated PCF is believed to gradually increase trust between individuals and organizations [42]. By contrast, contract breach yields various negative effects on the relationship between employees and organization [44]. For example, employees may lose trust in the organization, reduce their input to match their employer’s output, and thus consider quitting the organization [6]. Psychological contract breach generally exhibits a stronger effect on employees than the other aspects because prospect theory posits that people perceive loss as more harmful than the pleasure of gain [45]. Therefore, employers should understand mutual obligations and the extent to which employees perceive the former’s fulfillment of psychological contracts.

### 2.2. Psychological Contract Fulfillment and Task Performance

Task performance refers to an employee’s effectiveness in completing his/her core job or role-based responsibilities and is thus defined as in-role performance [46,47,48]. As an essential component of overall job performance, task performance reflects how an employee performs his/her required tasks [48]. Social exchange theory, which stresses reciprocity, provides a theoretical foundation to understand how employees respond to their perception of whether their psychological contracts have been fulfilled [12,49]. Psychological contract breach occurs when employees perceive a discrepancy between how they are actually treated and what they are promised [36]. Employees will not sufficiently fulfill their obligations to their employer when they feel shortchanged by their employer’s failure to fulfill its obligations [8,9]. Psychological contract breach can increase employees’ intention to turn over and decrease their level of organizational commitment and in-role and extra-role work performance [12]. By contrast, when employees perceive that their employers have actually provided more than what they have promised, such as larger pay raises, improved benefits packages, and additional opportunities for career development, they are likely to broaden and strengthen the social exchange relationship by increasing their contributions to the organization [50]. PCF is significantly related to employees’ attitudes and their behavior, with regard to job satisfaction, organizational commitment, organizational trust, absenteeism, turnover intention, performance, and organizational citizenship behavior [51,52,53,54,55]. In particular, the extent of PCF is positively related to employees’ in-role performance [49]. Within the service context, employees have shown a willingness to perform improved service behavior when they perceive that their employers or supervisors fulfill their obligations, and psychological contracts include transactional and relational terms, such as salary, working hours, job security, training opportunities, and a pleasant work environment [38]. Consequently, this study assumes positive relationships between PCF and gig workers’ task performance. The following hypotheses are proposed:

**Hypothesis** **1 (H1).** Transactional contract fulfillment is positively related to gig workers’ task performance.

**Hypothesis** **2 (H2).** Relational contract fulfillment is positively related to gig workers’ task performance.

### 2.3. The Mediating Effect of Organizational Identification

Organizational identification is defined as the extent to which employees identify themselves with their perceived representation of the organization or simply their perceived oneness with or belongingness to the organization [13]. When employees identify strongly with their organization, their needs for affection and affiliation are fulfilled [56,57,58]. Employees incorporate their employer’s identity into their own social identity when they identify themselves with the organization [10]. Masterson and Stamper (2003) mentioned that organizational identification accords with the belonging dimension of perceived organizational membership, regarding the perception that one has invested in becoming a member of the organization and a sense of perceived acceptance by the organization [59]. Thus, organizational identification is a critical component of the relationship between employees and the organization.

Within the context of psychological contracts, studies have revealed the complex and considerable effects of psychological contract breach and fulfillment on organizational identification. The organizational membership framework illustrates that psychological contract breach weakens employees’ perceptions of organizational membership because it is associated with their perception of unfulfilled needs [59,60]. The negative relationship between psychological contract breach and organizational identification has been empirically confirmed by numerous studies [10,61,62]. In particular, transactional psychological contract breach, involving economic or monetary terms and conditions, is found to exhibit a limited effect on organizational identification. Nevertheless, relational psychological contract breach, including socio-emotional elements, communicates symbolic messages about the negative standing of an employee within the organization, thereby hindering organizational identification [39]. Psychological contract breach discourages employees from investing in the organization, and their sense of belongingness is seriously eroded [63]. In contrast, PCF is an important driver of employee organizational identification [6]. PCF reflects employees’ need fulfillment via membership in the organization [59]. This concept can decrease emotional exhaustion and increase psychological well-being [64,65]. Fulfilled promises reduce employees’ uncertainty, thereby motivating organizational identification [6]. Although few studies have discussed PCF in the sharing economy context, part-time employees have responded to adjustments in their psychological contract in a similar manner as full-time employees [66]. Thus, this paper proposes positive relationships between PCF and a gig worker’s organizational identification in the sharing economy, including transactional and relational contract fulfillment. The hypotheses are developed as follows:

**Hypothesis** **3 (H3).** Transactional contract fulfillment is positively linked to gig workers’ organizational identification.

**Hypothesis** **4 (H4).** Relational contract fulfillment is positively linked to gig workers’ organizational identification.

Psychological contract breach can be construed by employees as unjust treatment and thus may be symbolic to some extent [39]. Restubog et al. (2008) found that organizational identification mediates the relationship between psychological contract breach and organizational citizenship behavior [39]. This mechanism underlying the relationship between psychological contract breach and its work outcomes has also been examined in the service literature [7,12,67]. For instance, when a hotel employee perceives that the hotel has violated the psychological contract by neglecting its promises, he/she will no longer possess a strong bond with the organization, thereby weakening his/her organizational identification and hindering job-related outcomes, including performance [7]. A study of ethnic minority employees’ employment relationship found that relational psychological breach both directly reduces ethnic minority employees’ organizational citizenship behavior and indirectly reduces their organizational identification [11]. Organizational identification represents an attachment in which the characteristics and success of the organization are incorporated into an individual’s self-concept [68]. Social identity theory posits that individuals act and behave in a manner that is consistent with the strength of their identification and thus enhance their self-esteem [69]. Individuals with a high level of organizational identification are less restricted in defining their job roles and engaging in activities that benefit their organization [70,71] and are more likely to increase their in-role performance and devote more effort toward their role than those with a low level of organizational identification [13,69,72]. Organizational identification serves as an important mediator between psychological contracts and employees’ in-role performance [17]. Individuals with a high perception of PCF perceive that the organization has satisfied their expectations in terms of providing economic or monetary rewards, offering socio-emotional support and fostering fairness. Employees’ perceived PCF can result in trust, loyalty, and organizational identification, which in turn motivate their organizational citizenship behaviors [9]. Gig workers in the sharing economy perceive a low level of belongingness, thereby potentially lowering their organizational identification. Transactional and relational psychological fulfillment may be positively linked to employees’ job satisfaction, security, organizational commitment, and identification, which may motivate them to improve their task performance [73]. Based on the literature discussed above, this paper proposes the following hypotheses:

**Hypothesis** **5 (H5).** Organizational identification is positively related to gig workers’ task performance.

**Hypothesis** **6a (H6a).** Organizational identification mediates the indirect relationship between transactional contract fulfillment and gig workers’ task performance.

**Hypothesis** **6b (H6b).** Organizational identification mediates the indirect relationship between relational contract fulfillment and gig workers’ task performance.

### 2.4. The Moderating Effects of Length of Service

Several terms are used to express the duration of service or employment, such as job tenure, organizational tenure, and length of service. [74,75,76]. Job tenure is defined as the length of time an employee performs the job or holds a position. Organizational tenure refers to the time an employee works for a particular organization [74]. Compared with job or organizational tenure, length of service is a more inclusive concept that does not necessarily involve employment relationships. Prior studies have investigated the moderating effect of employees’ tenure (or length of service) on the relationships between other variables. Organizational tenure moderates the relationship between job satisfaction and job performance [77]. In particular, the relationship between job satisfaction and performance is stronger for individuals with short organizational tenure than for those with long organizational tenure. Job tenure moderates the relationship between employees’ career adaptability and job contentment plateau, and this relationship is stronger for employees with a longer tenure than for those with a shorter tenure [78]. A meta-analysis revealed the moderating effect of employees’ tenure on the relationship between organizational commitment and job performance [79]. Organizational tenure also acts as a moderator of the relationship between affective organizational commitment and organizational citizenship behavior (OCB), and the strength of the commitment-OCB relation increases as organizational tenure increases [80].

Riketta (2005) conducted a meta-analysis and suggested that tenure is significantly related to organizational identification [72]. Highly tenured employees’ attitudes are more stable and less contingent upon PCF than those of shortly tenured employees [19]. Being accepted as a contributing member in the organization is the major concern for a new employee [81]. The organization should send “signals” to an employee to communicate acceptance. These “signals” include sharing organizational secrets, initiation rites, and increased autonomy, which reflect trust and a close relationship. Moreover, the evidence of successful job performance, such as appraisal scores and rewards (e.g., improvement in salary), are the most important early signals for new employees to perceive acceptance [77]. The studies cited above conclude that relational and transactional motivations can increase perceived acceptance and organizational identification for newcomers. Within the sharing economy context, gig workers perceive weak organizational identity. However, individuals adapt to organizational values as their tenure increases [82]. Thus, we believe that PCF has more significant effects on the organizational identification of gig workers who have a short length of service in the current company than those who have a long length of service. The hypotheses are as follows:

**Hypothesis** **7.** Transactional contract fulfillment (H7a) and relational contract fulfillment (H7b) have stronger effects on the organizational identification of gig workers who have a low length of service than those who have a high length of service.

Organizational tenure has been shown to moderate the reciprocal relationships between psychological contracts and job outcomes [19]. The behavior of employees with short tenure is driven primarily by reciprocity norms, whereas long-tenured employees’ behaviors are driven primarily by affective factors, including loyalty [37,79]. A new employee’s investment in his/her job is based primarily on inducement (e.g., attractive reimbursement) that they receive from the organization. By contrast, the links between employees’ efforts and their rewards received are relatively weaker for long-tenured employees than for short-tenured employees [20]. When employees are engaged in their work and decide to stay within the organization as their tenure increases, they perceive higher obligations to their relationships within the organization and are more likely to contribute to organizational effectiveness than do those who are not engaged in their work and unwilling to remain in the organization [19]. Long-tenured employees’ performance is likely related to their relational PCF, which consists of trust, good faith, and exchange of intangible constructs with long time frames [34]. Based on the cited studies, this paper assumes that the relationships between different types of psychological contract fulfillment and gig workers’ task performance are moderated by the length of service in separate ways. Therefore, the following hypothesis is proposed.

**Hypothesis** **8.** The relationship between transactional contract fulfillment and task performance is stronger for gig workers who have a short length of service than for those with a long length of service (H8a), whereas the relationship between relational contract fulfillment and task performance is stronger for gig workers who have a long length of service than for those with a short length of service (H8b).

### 2.5. Research Significance and Conceptual Model

**Figure 1:**
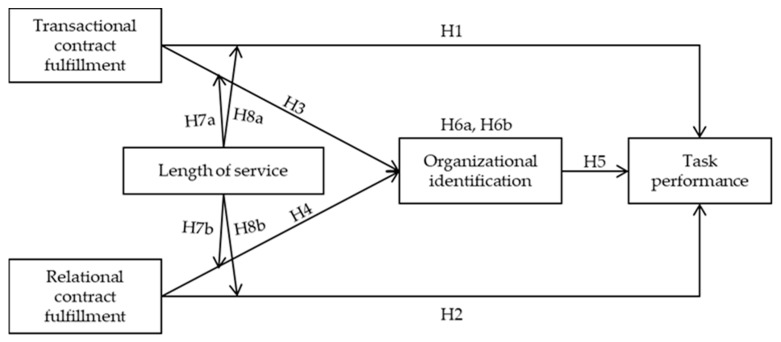
Research model.

As previously mentioned, PCF is an important driver of the organizational identification of employees and their performance in traditional employment settings [6,12,49]. However, in a sharing economy, gig organizations hire independent workers on short-term contracts to accomplish temporary tasks, generally by connecting these workers to customers through their platform. As they work separately from their organizations, gig workers perceive a weak affiliation with their employers. In this unique context, whether and how PCF motivates gig workers’ job outcomes are yet to be investigated. Moreover, whether gig workers join the platform primarily for economic purposes or for sustaining long-term relationships and whether the fulfilment of a transactional psychological contract is as significant as the fulfilment of a relational psychological contract in predicting the performance of gig workers are not supported by any clear evidence. To answer these questions, this research aims to identify the relationships among the constructs in the following conceptual model (shown in Figure 1) that is generated based on the aforementioned hypotheses:

## 3. Methods

### 3.1. Measures

A survey questionnaire was constructed based on the model. To measure each construct, questions were adopted from validated instruments used in previous studies and modified to fit our research context.

Transactional contract fulfillment and relational contract fulfillment were assessed using the measures adopted from Bal et al. [23], Wu and Chen [38], and Turnley et al. [49]. Although the transactional contract consists of both adequate monetary/economic compensation and non-monetary aspects, including reasonable working conditions and guarantees of employment [11], the latter dimensions are inapplicable to gig employment. Moreover, the dimensions of pay and a supportive employment relationship have been used in the research by Turnley et al. (2003), both because they anchor the ends of the transactional-relational continuum that has been discussed in some prior studies [9,34] and because of their salience to employees [49]. Thus, this research measures the transactional psychological contract by focusing mainly on the economic aspects, namely, income and profit sharing. Specifically, transactional contract fulfillment was measured by using four items, such as “I am receiving competitive wages compared with people working for other sharing economy companies” and “My income is tied to the level of my performance.” Similarly, relational transactional contract fulfillment was measured by using four items, such as “I am always treated fairly and impartially” and “The company provides many training and development opportunities for me.” Following the approaches used by Bal et al. [23], Wu and Chen [38], and Turnley et al. [49], the respondents in this research were asked to answer PCF-related questions on a five-point Likert scale ranging from 1 (strongly disagree) to 5 (strongly agree).

Organizational identification was measured by using four items adapted from Epitropaki [63], Li et al. [7], Zagenczyk et al. [10], and Lu et al. [17]. A sample question is “I feel proud to work for this organization.” Meanwhile, the items for measuring task performance were adapted from Bal et al. [23], Turnley et al. [49], and Sobaih et al. [83]. A sample question is “I can always fulfill all the responsibilities specified in my task description.” By adopting the approach used by Zagenczyk et al. [10], Bal et al. [23], and Turnley et al. [49], the responses to the questions about organizational identification and task performance are rated from 1 (strongly disagree) to 5 (strongly agree). Table 1 lists the items for all constructs.

### 3.2. Sample and Procedure

Didi chuxing (Didi), a ride-hailing platform, was selected as the research object because it is one of the most representative sharing economy platforms in China, and its drivers constitute the largest gig worker group in China. The data were collected through an interview survey among 223 Didi drivers from March to September 2019. To avoid interrupting the drivers’ work, our research team members interviewed Didi drivers during their breaks and subsequently recorded their answers. A flag-down fare was paid to each driver after the interviews. Table 2 reports the participant demographics. A total of 97.2% of the participants were male, whereas only 2.2% were female. This ratio indicates that the ride-hailing industry is very male-dominated. Most of the participants were more than 25 years old (96.8%) and had a high school education or below (74.9%). In terms of the length of service for the current company, 48% of the respondents answered less than 1 year, whereas 52% of them had more than 1 year of experience. Most of the participants relied on this job as a main source of income, given that 84.3% of them worked for more than eight hours per day.

### 3.3. Analyses of Validity and Reliability

A confirmatory factor analysis (CFA) with AMOS 21.0 was conducted to examine the discriminant validity of the main variables. The four-factor model that includes transactional contract fulfillment, relational fulfillment, organizational identification, and job performance was initially assessed. The fit indices show that this model is quite acceptable (*χ^2^*/df = 1.350, GFI = 0.939, RFI = 0.917, TLI = 0.977, CFI = 0.982, RMR = 0.057, RMSEA = 0.040). Subsequently, four alternative models were compared with the proposed four-factor model. Table 3 reveals that the values and significance of Δχ^2^ indicate that the proposed four-factor model has better model fit indices than the other four alternative models, and the discriminant validity of the main constructs was preliminarily examined.

The reliability and convergent validity of the variables were also examined. Table 4 shows that all factor loadings in the measurement model exceeded 0.7, except for OI4 (factor loading is 0.691) and JP1 (factor loading is 0.662), which are also extremely close to 0.7. Moreover, all values of reliability (Cronbach’s α) and composite reliabilities are greater than 0.7, thereby demonstrating the convergent validity of our measures [84].

Table 5 indicates that the square roots of all average variance extracted (AVE) are higher than the correlations between the target variable and any of the other variables, which confirms the discriminant validity of the variables.

### 3.4. Common Method Deviation Test

To prevent common method deviation in the data, the Harman single-factor test was carried out. The results show that the interpretation of the first factor of the unrotated exploratory factor analysis is 31.12%, which fails to exceed the 40% recommendation of Podsakoff et al. (2003) [85]. Furthermore, the CFA results show that the one-factor model (*χ^2^*/df = 11.582, GFI = 0.527, RFI = 0.288, TLI = 0.307, CFI = 0.406, RMSEA = 0.218) exhibits a worse fit than our measurement model. Thus, common-method bias is not an issue in this study.

## 4. Hypotheses Test

### 4.1. Summary of the Path Analysis Results

**Figure 2:**
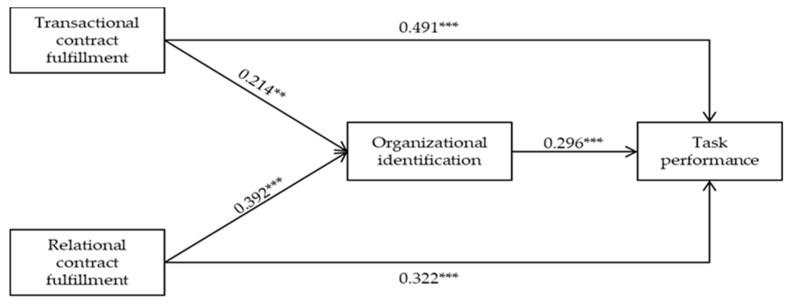
Path coefficient of the hypothesis model. **p < 0.01; ***p < 0.001.

A structural equation modeling analysis is conducted on AMOS 21.0 to test the hypotheses proposed in this study. The result presents a good model fit (χ^2^/df = 1.350, GFI = 0.939, RFI = 0.917, TLI = 0.977, CFI = 0.982, RMR = 0.057, RMSEA = 0.040; coincidentally, the values of the fit indices are similar to the measurement model). Figure 2 illustrates the path coefficients between each pair of variables in the structural model.

Figure 2 reflects that the path coefficient between transactional contract fulfillment and gig workers’ task performance (β = 0.491, *p* < 0.001) is positively significant, thereby supporting Hypothesis 1. The path coefficient between relational contract fulfillment and task performance is also significant (β = 0.322, *p* < 0.001), which supports Hypothesis 2. The path coefficients between transactional contract fulfillment and organizational identification (β = 0.214, *p* < 0.01) and between relational contract fulfillment and organizational identification (β = 0.392, *p* < 0.001) are also significant, thereby validating Hypothesis 3 and Hypothesis 4. The significant positive path coefficient between organizational identification and task performance (β = 0.296, *p* < 0.001) supports Hypothesis 5.

Previous studies have suggested that PCF can lead to employees’ organizational identification, thereby motivating their organizational citizenship behaviors. Given that high-quality task performance is considered an essential aspect of organizational citizenship behavior, this study examines how organizational identification mediates the relationship between PCF and task performance. A mediating effect analysis was conducted based on the bootstrap method (1000 bootstrap samples, 95% PC). Table 6 reveals that transactional contract fulfillment has a relatively weak (compared with direct effect: β = 0.491, *p* < 0.01) but significant indirect effect (β = 0.063, *p* < 0.05) on task performance via organizational identification, thereby partially supporting Hypothesis 6a. In addition, relational contract fulfillment has a significant indirect effect (β = 0.116, *p* < 0.01) on task performance via organizational identification, accepting Hypothesis 6b.

### 4.2. Summary of the Moderating Effect Analysis Results

**Figure 3:**
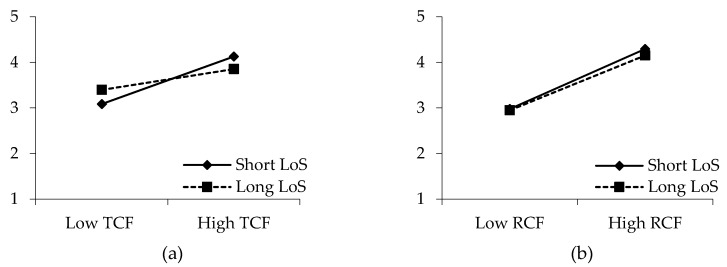
Moderating effects of the length of joining in the company (LoS) between **(a)** transactional contract fulfillment (TCF) and organizational identification (OI) and **(b)** relational contract fulfillment (RCF) and organizational identification (OI).

Hypotheses 7a and 7b propose the moderating effects of drivers’ tenure in the current company on the relationships between PCF and organizational identification. To test these hypotheses, this study conducted multigroup path analyses after dividing the samples into two groups based on the mean of the length of service (LoS). The findings reveal that the change in the value of χ^2^ is statistically significant (Δχ^2^ = 4.047, *p* < 0.05) when the path is constrained between transactional contract fulfillment and organizational identification. The slopes in Figure 3a indicate that transactional contract fulfillment can more effectively boost the organizational identification of drivers who joined Didi within the year (β = 0.336, *p* < 0.01, N = 107) compared with those who joined Didi more than a year earlier (β = 0.014, *p* > 0.05, N = 116). Thus, hypothesis 7a is supported. However, when the path between relational contract fulfillment and organizational identification is constrained, the change in the χ^2^ value is insignificant (Δχ^2^ = 0.024, *p* > 0.05). Figure 3b shows that the difference between the two slopes is unremarkable. Therefore, hypothesis 7b is rejected.

**Figure 4:**
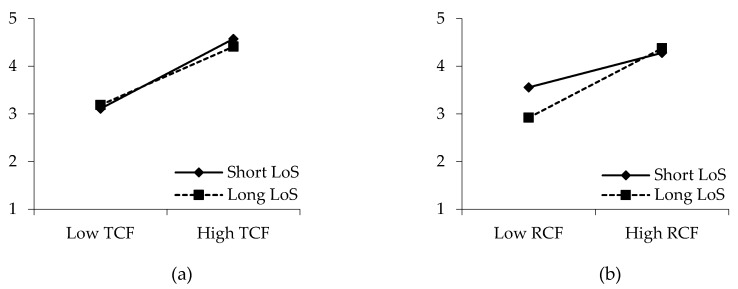
Moderating effects of the length of joining in the company (LoS) between **(a)** transactional contract fulfillment (TCF) and task performance (TP) and **(b)** relational contract fulfillment (RCF) and task performance (TP).

Hypotheses 8a and 8b propose the moderating effects of drivers’ tenure in the current company on the relationships between PCF and task performance. The results reveal that the change in the *χ^2^* value is nonsignificant (Δ*χ ^2^* = 0.001, *p* > 0.05) when the path between transactional contract fulfillment and task performance is constrained. Figure 4a demonstrates that the difference between the two slopes is insignificant. Therefore, hypothesis 8a is rejected. In contrast, when the path between relational contract fulfillment and task performance is constrained, the change in the *χ^2^* value is significant (Δ*χ^2^* = 3.952, *p* < 0.05). The slopes in Figure 4b indicate that drivers who had worked with Didi for more than a year are more motivated to perform high-quality work (β = 0.461, *p* < 0.001, N = 116) by relational contract fulfillment than drivers who had worked for Didi for less than a year (β = 0.186, *p* > 0.05, N = 107). Therefore, hypothesis 8b is validated.

## 5. Discussion

PCF is identified as a remarkable perspective to understand individuals’ organizational citizenship behavior. Derived from social exchange theory, psychological contracts are the foundation of the relationship between individuals and the organization [29]. The impact of PCF on employee performance under traditional employment has been widely acknowledged for many years. Drawing on previous literature, this study proposes a framework for examining whether and how PCF enhances the organizational identification and task performance of gig workers in a sharing economy context.

The results of this study indicate that transactional and relational PCF can contribute to gig workers’ task performance. The results are consistent with previous studies in the field of organizational behavior. For example, PCF, including its economic and relational aspects, is positively related to the in-role performance and other organizational citizen behaviors of individuals who are employed in full-time positions [49]. Moreover, this study reveals the mediating mechanism of organizational identification between PCF and gig workers’ task performance. The mediating role of organizational identification has been discussed in a few previous studies. For example, a study targeting hotel employees has investigated the mediating effect of organizational identification between psychological contract breach and job performance and found that psychological contract breach can result in low job performance by weakening employees’ organizational identification [7]. Another study focused on task performance and found that organizational identification mediates the indirect relationship between relational psychological contract and service-oriented in-role performance but not transactional psychological contract [17]. By contrast, the result of this study indicates that not only transactional PCF but also relational PCF indirectly influence the task performance of gig workers via organizational identification. Compared with full-time employed workers, gig workers are isolated or not physically present in their organizations and may perceive a lower organizational identification. These findings suggest that organizational identification forms as a result of relational PCF and becomes a crucial motivator for gig workers.

Drawing on the concept of organizational tenure, this study proposes and examines the moderating role of length of service on the relationship between PCF and gig workers’ attitudinal and behavioral outcomes. The results indicate that the influence of transactional contract fulfillment on organizational identification is stronger for newcomers who joined the company within the year than for those who joined the company earlier. In contrast to the influence of relational contract fulfillment, transactional contract fulfillment can increase task performance for all gig workers, regardless of their tenure in the company. Relational contract fulfillment has a stronger effect on long-length gig workers’ task performance than newcomers. These findings are consistent with the views of several previous studies. The effects of monetary-related items are not moderated by old workers’ identity [86]. However, the relational psychological contract consists of trust and good faith, sustained reciprocation, and the exchange of intangible constructs that require long time frames to develop [34]. This notion suggests that the contributions of long-tenured employees are motivated by their history of relationship with the organization [42]. The results of this present study reflect the aforementioned view, which indicates that relational PCF is an important predictor for the task performance of gig workers who have a long length of service in the current company.

## 6. Implications and Limitations

### 6.1. Theoretical Implications

This study enriches the theoretical studies about the sharing economy from the perspective of gig employment. Certain previous studies have investigated gig workers’ organizational trust, engagement, job satisfaction, and other job outcomes, but few studies have discussed organizational identification and performance [3,4,5]. Given that gig workers are employed by companies in the sharing economy without a long-term employment relationship, they may perceive that they have low job security. This paper proposes that PCFs are important drivers for gig workers to engage in their work and generate effective commitment to the company in which they currently work. The study’s findings suggest that psychological contract and organizational identification should be highly regarded and added to the theoretical framework of gig employment research.

First, this study focuses on the driver of gig workers’ task performance and explores the effect of PCF. Although previous studies have examined the relationship between PCF and employees’ performance, they generally focus on job performance and the performance of employees’ organizational citizenship behaviors, which are relatively broad aspects of employees’ behavior [38,83]. In the sharing economy, individuals under gig employment work more independently and are more likely to concentrate on their own tasks or in-role performance than other organizational behaviors. Thus, the present study specifically employs task performance as the outcome of PCF and examines the positive relationship between them. This finding theoretically provides a new approach regarding the psychological contract to predict gig workers’ task performance.

Second, PCFs in this study, including transactional and relational PCFs, exhibit positive effects on gig workers’ organizational identification, which indicates that organizational identification is also an important concept in gig employment. Moreover, organizational identification plays a mediating role between PCFs and gig workers’ task performance. Although this mechanism varies across different contexts [7,17], this study demonstrates that the relationships of transactional and relational PCFs with task performance can be mediated by organizational identification. In conclusion, the results of the present study provide theoretical support for the direct path from PCF to gig workers’ task performance and the indirect path between them via organizational identification.

Lastly, drawing on the moderating role of organizational tenure in the relationship between PCF and employee work outcomes [21,66], this study examines the role of service length of gig workers for their current companies. Results show that the influence of transactional PCF on organizational identification is stronger for gig workers who have a short service length than for those with a long service length, whereas the effects of relational PCF do not differ between these two groups. However, relational PCF has a stronger influence on the task performance of gig workers with a long service length, whereas transactional PCF has similar effects on the task performance of both groups. These findings imply that new workers in a sharing economy can form organizational identification through the recognition they perceive from the rewards provided by their companies. By contrast, the contribution of relational PCF on performance is more significant for gig workers who have worked for their current company for a long period. The moderating mechanism of service length examined in this study offers a new perspective to understand the organizational citizen behavior of gig workers and a theoretical support for studying different groups of individuals in a sharing economy.

### 6.2. Practical Implications

This study has several managerial implications. First, it is empirically evident that the psychological contract will increase individuals’ satisfaction with the organization, thereby inducing organizational identification and enhancing their performance. In contrast, a breach of psychological contract is construed by individuals as unjust treatment and thus results in organizational misidentification [10]. In the sharing economy context, relationships between gig workers and the organization are relatively weaker than those in traditional employment situations. Psychological contract breach will exhibit a more significant negative effect on gig workers’ organizational identification. By contrast, if gig workers perceive that the organization has actually exceeded their expectations (e.g., through unexpectedly high income, bonuses, training or development opportunities, or other benefits), then they may endeavor to reciprocate by performing organizational citizenship behaviors, particularly high-quality performance. Thus, companies should exert effort to understand gig workers’ psychological contracts with companies and to fulfill their psychological contracts, including transactional and relational psychological contracts.

Second, organizational identification reflects the affective commitment and attachment to the organization, and its characteristics and success are incorporated into an individual’s self-concept [68]. Hence, organizational identification is perceived to have important implications for individuals’ behaviors [17]. In the sharing economy, companies serve as platforms that meet consumers’ needs as well as those of gig workers who are not formally employed in companies. Thus, gig workers can hardly perceive organizational identification. Companies must recognize the significance of gig workers’ organizational identification because they are the capital and determinant of their organization’s success. When gig workers identify with the company, their self-interest and the company’s interest become enmeshed, and the organization’s achievements become a personal achievement. Gig workers with a high level of organizational identification are less restricted in defining their job roles and engage in activities that benefit the company than those with low organizational identification. That is, if gig workers perceive a strong organizational identification, they are likely to increase their performance and direct effort applied to their roles to reinforce the organization’s interests and their own self-esteem. According to the findings of this study, companies should enhance gig workers’ organizational identification by fulfilling both their transactional and relational psychological contracts to the company.

Third, this present study finds that transactional PCF is more important for gig workers who have a short length of service to form organizational identification than for those with a long length of service. This finding indicates that transactional contract fulfillment (e.g., competitive income or fair profit sharing) is regarded by gig workers as a signal of recognition of their performance and acceptance by the organization. This instance helps gig workers to perceive a close relationship with the organization and consequently incorporate the organization’s identity into their own social identity. Owing to organizational identification, gig workers may enhance their commitment and aptitude to perform organizational citizenship behavior. Simultaneously, relational psychological fulfillment has a greater influence on the task performance of gig workers who have worked for the company for relatively long than on that of newcomers. This outcome occurs because gig workers who have a long service experience for the current company perceive a stable relationship with the company, and their performances are not only driven by economic factors but also motivated by affective factors. They have invested both transactional and relational effort into the company. Given that the give-and-take only can hardly be mapped precisely based on transactional exchange, companies should be concerned about the relational needs of their senior workers and treat them as important members of the company by providing them with additional training to learn new skills, opportunities to attend company’s activities, and care and respect from the management. When gig workers perceive that they are valued by the company, they will self-identify as members of the company and improve their performance.

### 6.3. Limitations and Future Research

Along with its contributions, this study has certain limitations. First, the survey data reveal that most of Didi’s drivers work for the company for more than 8 hours per day, whereas less than 20% of them work for less than 8 hours per day. Most drivers perform this work as a full-time job, while others do so as a part-time job. Thus, gig workers’ organizational identification and task performance may depend on whether they view this job as their career. Second, by assuming that gig workers generally work independently, the present study focuses mainly on the effect of PCF and organizational identification on their task performance. When gig workers’ psychological contracts are fulfilled and they identify themselves as members of the company, they may perform other aspects of organizational citizenship behavior to benefit the organization, such as organizational loyalty, sportsmanship, altruism, and civic virtue. Third, the present study investigates the influences of PCF. However, previous studies have suggested that psychological contract breach has strong effects on employees’ attitudinal and behavioral outcomes, whereas organizational identification can mediate these effects under the traditional employment context. Therefore, the mediating effects of organizational identification between psychological contract breach and job outcomes can be examined in the gig employment context in the future. Fourth, this study successfully interviewed only 223 Didi drivers because it was difficult to find drivers who were willing to participate in our investigation. Considering the rapid growth of the sharing economy and the significance of studying gig workers, enhanced approaches to motivate gig workers’ participation should be explored.

## 7. Conclusions

This study theoretically proposes and empirically examines whether PCF enhances gig workers’ organizational identification and performance. The results show that PCF positively affects gig workers’ task performance. Organizational identification partially mediates the relationship between PCF and task performance. Moreover, the moderating effects of length of service on the relationships between PCF and organizational identification, as well as task performance, were examined. The results show that transactional contract fulfillment is more effective for facilitating the organizational identification of drivers who have a low length of service for Didi than for those with a short length of service in such companies. On the other hand, drivers who have many years of service in Didi are more likely to be motivated by relational contract fulfillment to perform high-quality work than those with few years of service in the company. Based on these findings, several theoretical and practical implications and directions for future work are proposed.

## Figures and Tables

**Table 1:** Research constructs and measurements.

Construct	Measurement Items	Sources
Transactional contract fulfillment (TCF)	TCF1: Competitive income compared with people working for other sharing economy companies	[23,38,49]
	TCF2: Fair profit sharing for responsibilities that the company and I have in the job	
	TCF3: Income tied to the level of my performance	
	TCF4: Income increase to maintain my standards of living	
Relational contract fulfillment (RCF)	RCF1: The extent to which I am treated fairly and impartially	
	RCF2: The extent to which I am treated with respect	
	RCF3: The amount of training and development provided by the company	
	RCF4: The amount of all kinds of support I received from management	
Organizational identification (OI)	OI1: I feel proud to work for this organization	[7,10,17,63]
	OI2: When someone criticizes this organization, I would feel embarrassed	
	OI3: Someone’s praise to this organization feels like a personal compliment	
	OI4: I view the organization’s success as my success	
Task performance (TP)	TP1: I fulfill all the responsibilities specified in my task description	[23,49,83]
	TP2: I pay attention to detail and avoid making mistakes	
	TP3: I look for improved ways to accomplish the assigned work	

**Table 2:** Demographic characteristics of the survey participants (*n* = 223).

Demographic Profile	Categories	Frequency	Percent (%)
Gender	Male	218	97.8
	Female	5	2.2
Age	≤25	7	3.1
	26−35	100	44.8
	36−45	64	28.7
	>45	52	23.3
Education	High school or below	167	74.9
	University/College	56	25.1
Length of service for current company	≤1 year	107	48
	1−2 years	65	29.1
	2−3 years	36	16.1
	>3 years	15	6.7
Hours of work per day	≤4 hours	7	3.1
	4−6 hours	7	3.1
	6−8 hours	21	9.4
	>8 hours	188	84.3

**Table 3:** Confirmatory factor analysis results of alternative models.

Model	*χ^2^*	df	Δ*χ^2^*	GFI	RFI	TLI	CFI	RMSEA
Four-factor model TCF, RCF, OI, TP	113.428	84		0.939	0.917	0.977	0.982	0.040
Alternative three-factor model TCF + RCF, OI, TP	607.681	87	494.253 ***	0.679	0.571	0.679	0.608	0.164
Alternative two-factor model TCF + RCF, OI + TP	744.865	89	631.437 ***	0.633	0.486	0.517	0.591	0.182
Alternative two-factor model TCF + RCF + OI, TP	748.456	89	635.028 ***	0.602	0.483	0.515	0.589	0.183
Single-factor model TCF + RCF + OI+ TP	1042.344	90	928.916 ***	0.527	0.288	0.307	0.406	0.218

Note. *** *p* < 0.001.

**Table 4:** Test results of internal reliability and convergent validity.

Construct	Items	Cronbach’s α	Convergent Validity		
			Factor Loading	Composite Reliability	Average Variance Extracted
TCF	TCF1	0.872	0.733	0.873	0.633
	TCF2		0.808		
	TCF3		0.823		
	TCF4		0.816		
RCF	RCF1	0.876	0.863	0.877	0.642
	RCF2		0.747		
	RCF3		0.785		
	RCF4		0.806		
OI	OI1	0.865	0.786	0.867	0.622
	OI2		0.822		
	OI3		0.846		
	OI4		0.691		
TP	TP1	0.750	0.662	0.754	0.506
	TP2		0.710		
	TP3		0.758		

*χ^2^*/df = 1.350, GFI = 0.939, RFI = 0.917, TLI = 0.977, CFI = 0.982, RMR = 0.057, RMSEA = 0.040.

**Table 5:** Mean, standard deviation, and correlation matrix.

Variables	Mean	SD	TCF	RCF	OI	JP
TCF	3.370	0.972	0.796			
RCF	3.436	1.032	−0.100	0.801		
OI	3.693	0.924	0.152 *	0.330 **	0.788	
TP	3.963	0.831	0.413 **	0.303 **	0.399 **	0.711

Note. * *p* < 0.05, ** *p* < 0.01; the diagonal line of the correlation matrix represents the square root of AVE.

**Table 6:** Mediating effects of organizational identification.

Effect	Path	Standardized Estimate (β)	Lower Bound	Upper Bound	*p*
Total effect	TCF- > TP	0.554	0.429	0.680	0.002
Direct effect	TCF- > TP	0.491	0.368	0.611	0.002
Indirect effect	TCF- > OI- > TP	0.063	0.014	0.124	0.014
Total effect	RCF- > TP	0.439	0.299	0.591	0.002
Direct effect	RCF- > TP	0.322	0.172	0.483	0.003
Indirect effect	RCF- > OI- > TP	0.116	0.054	0.197	0.002
